# Measuring Chess Experts' Single-Use Sequence Knowledge: An Archival Study of Departure from ‘Theoretical’ Openings

**DOI:** 10.1371/journal.pone.0026692

**Published:** 2011-11-16

**Authors:** Philippe Chassy, Fernand Gobet

**Affiliations:** 1 Institute of Medical Psychology and Behavioral Neurobiology, University Hospital Tübingen, Tübingen, Germany; 2 Department of Psychology, Brunel University, Uxbridge, United Kingdom; University of Sheffield, United Kingdom

## Abstract

The respective roles of knowledge and search have received considerable attention in the literature on expertise. However, most of the evidence on knowledge has been indirect – e.g., by inferring the presence of chunks in long-term memory from performance in memory recall tasks. Here we provide direct estimates of the amount of monochrestic (single use) and rote knowledge held by chess players of varying skill levels. From a large chess database, we analyzed 76,562 games played in 2008 by individuals ranging from Class B players (average players) to Masters to measure the extent to which players deviate from previously known initial sequences of moves (“openings”). Substantial differences were found in the number of moves known by players of different skill levels, with more expert players knowing more moves. Combined with assumptions independently made about the branching factor in master games, we estimate that masters have memorized about 100,000 opening moves. Our results support the hypothesis that monochrestic knowledge is essential for reaching high levels of expertise in chess. They provide a direct, quantitative estimate of the number of opening moves that players have to know to reach master level.

## Introduction

A classic debate in the research into expertise concerns the respective roles of knowledge and search. Early work by de Groot [1] on chess emphasized the importance of knowledge, with a concomitant de-emphasis of the role of search: in a problem-solving task, important skill differences in perception and understanding were already apparent after 5 s, while analyses of the structure of search (e.g., depth of search, width of search) hardly found any differences between players of different skill levels. While later research has considerably increased our understanding of how knowledge mediates expertise, there is a type of knowledge that has received surprisingly little attention: monochrestic (single-use) knowledge, which tends to be rote knowledge. Is monochrestic knowledge important in attaining high levels of skill in certain fields? If we except domains where rote memory is the object of the skill (e.g., memorizing as many digits of π as possible), little research has been carried out to answer this question.

The aim of this paper is to address this question with chess, a domain that has much contributed to our scientific understanding of expertise. We first briefly review evidence showing the importance of search in chess expertise. We then present evidence supporting the role of non-declarative and declarative knowledge. This will bring us to the central question of this paper – the role of monochrestic knowledge in expert behavior. This issue will be investigated by exploring the extent to which players of different skill levels know sequences of moves in the opening phase of the game. An analysis of a large number of games played by players of four different levels will allow us to estimate the average depth of the known opening sequences as a function of skill. Based on these results and other data in the literature, we then provide mathematical models aimed at estimating the number of opening moves that are known by players of different levels.

### Evidence for search

Contrasting with the original conclusion of de Groot [1], considerable evidence has demonstrated skill differences in search. As noted by Holding [2], most of the analyses carried out by de Groot compared grandmasters with candidate masters (players three standard deviations [i.e., 600 Elo points] above the level of average players; see Materials and Methods), and this might have masked some of the differences. As a matter of fact, when more recent research used weaker players in addition to players at or above expert level, it consistently demonstrated skill differences in search behavior [3]–[5], with stronger players searching more. Interestingly, in some cases very strong players (international masters and grandmasters) searched much less than masters [6]. A possible reason for these discordant results might be that different investigators used positions of different levels of complexity. To test this hypothesis, Campitelli and Gobet [7] used very complex chess positions and found a strong skill effect for measures such as depth of search and number of nodes generated. Importantly, these variables had much higher absolute values (sometimes by a factor of ten) than in previous research. Putting together their results and previous literature, Campitelli and Gobet concluded that chess experts adapt their search algorithm as a function of the demands of the task: when facing simple positions and/or under time pressure, they mostly rely on pattern recognition made possible by their long-term memory knowledge; when facing complex positions and with enough thinking time, they carry out extensive search. This conclusion has been supported by recent experimental research [8], [9]. It is also consistent with a computer model, based on the template theory (see below), which shows how pattern recognition and search interact in skilled problem solving [10].

### Evidence for non-declarative knowledge

De Groot's [1] conclusion on the role of knowledge has been supported by later research. Analyzing the way players grouped pieces in a recall task (where the target position was presented for only 5 s) and in a copy task (where the position remained in view of the players), Chase and Simon found that a master used larger groupings than weaker players, a result that has been replicated several times [11], [12] and is thus beyond doubt. Chase and Simon explained these results with their “chunking” theory, which proposes that, with practice and study, individuals in chess and other domains acquire a large number of perceptual chunks (small groups of domain-specific information). These chunks help in a recall task, because groups of pieces rather than individual pieces can be stored in short-term memory. They also help in a problem-solving task, because some of the chunks are linked to potentially useful information, such as what kinds of moves are likely to be good in a given type of position. In line with these assumptions, Bilalić and colleagues [9] showed that players specialized in specific *openings* (the first moves of the game) performed much better (one standard deviation in skill) when dealing with positions from these types of opening, both in a recall and problem solving task, than when facing positions from openings they did not play. Thus, a grandmaster would play only at the level of a master when taken out of her domain of specialization.

Chase and Simon proposed that chunks encode relatively small amounts of information – a maximum of 5–6 pieces in chess. However, later research on problem solving, memory, and classification tasks [1], [13], [14] has uncovered clear evidence that chess experts use larger and higher-level representations as well. Cooke and colleagues [15] manipulated the type of board descriptions provided in a memory recall task, and found that such representations helped recall only if they were provided before (and not after) the presentation of the position to remember. Evidence for larger representations was also found in a particularly demanding task where not just one but several briefly presented positions had to be remembered simultaneously [15], [16]. Together, these results led to a revision of the chunking theory [17] with the template theory [16]. Template theory proposes that chunks used frequently by individuals become “templates”, a type of schema, which consists both of a *core* with constant information and *slots* where variable information can be stored. Note that chunks and templates are considered as non-declarative, as neither is available for conscious inspection.

### Evidence for declarative knowledge

In line with commonly-held views in chess circles, Holding [18] argued that chess experts have considerable declarative knowledge of chess openings, principles, strategies and tactics, and even entire games. This view was supported by a questionnaire study [19], where it was found that verbal chess knowledge accounted for 48% in variance in skill. However, a limit of this study was that there were only three players at the level of master and above, which means that conclusions cannot be drawn about highly skilled players. Charness [20] used books on chess opening (more specifically, the five-book series *Encyclopedia of Chess openings* [21]) to estimate the number of opening moves that experts know – a kind of declarative knowledge. Assuming that players know three or four systems with both white and black, he concluded that grandmasters know about 1,200 distinct opening sequences. Charness also discussed the knowledge that players have about middle games and endgames, although quantitative estimates turned out to be elusive. More recently, the role of declarative knowledge has been supported by an online chess test [22]. In addition to a choose-a-move task, a motivation questionnaire, a predict-a-move task, and a recall task, this test contained a short questionnaire about verbal knowledge, comprising fifteen four-alternative multiple-choice questions (these questions were partly adapted from [19]). The questionnaire accounted for 30% of variance in skill.

### Quantifying chess knowledge

While the considerable research on chess expertise has shown that both knowledge and search play an important role in expert behavior, a number of issues are unsettled. Particularly striking is the lack of *direct quantitative evidence* on the amount of knowledge held by masters. Although the research on chunking has generated a substantial amount of empirical data and has led to several detailed computational models, it has produced only fairly rough estimates of the number of chunks necessary to reach grandmaster level. Simon and Gilmartin [23] proposed a range from 10,000 to 100,000 chunks, while Gobet and Simon [24] proposed as many as 300,000 chunks. These estimates were also indirect, as they were made from computer simulations of the recall task. While compelling, the evidence for high-level representations is rather unsystematic and has not led to quantitative estimates of the amount of knowledge possessed by experts. Questionnaires on declarative knowledge have only been used rarely; furthermore, it is unclear how the results they provide could lead to quantitative estimates. Finally, Charness's [20] research was tentative and based on the knowledge contained in books, and thus only indirect inferences can be made about the knowledge held by chess players.

Estimating the size of knowledge mastered by an expert is obviously a difficult endeavor. There is first the difficulty of measuring procedural knowledge. However, even if the focus is on declarative knowledge, “extracting” this knowledge poses considerable difficulties, as has been known for decades in the fields of expert systems and knowledge engineering [25], [26]. In addition to the difficulty and cost of convincing grandmasters to disclose their knowledge and transcribing verbal protocols, it would be unclear what had been omitted from them.

### Polychrestic and monochrestic knowledge in chess

We introduce a crucial distinction between two types of knowledge: polychrestic and monochrestic. (These terms come from the ancient Greek μόνος (single), πολύς (many), and χρηστός (useful). We thus distinguish monochrestic knowledge – i.e., with single use – from polychrestic knowledge – i.e., with multiple uses.) *Polychrestic knowledge* refers to knowledge that can be used in different situations, possibly with changes to adapt it from case to case; in chess, this includes principles, strategies, and tactical motifs. This knowledge is encoded both declaratively and non-declaratively using chunks and templates. Polychrestic knowledge has been the focus of most previous research. *Monochrestic knowledge* refers to knowledge that can be applied to only one single situation. It is typically knowledge acquired from rote learning. Chess offers a perfect example of such knowledge (called *theoretical knowledge* in chess circles): the knowledge of moves in the first phase of the game (“openings”). Since all this knowledge is associated to the initial position of the game, it “unfolds” from it: openings are learned as sequences of moves, and each move in a given sequence has a precise function that cannot be generalized to other sequences. By learning these sequences, players learn the best set up of their pieces as a function of the opponent's reactions. Thus, the number of theoretical moves known by players is informative about the amount and depth of monochrestic knowledge necessary for reaching expert level.

Becoming a chess master requires a detailed knowledge of chess openings, as the outcome of a game can be decided by a single bad move at the beginning of a game. In openings, the overall aims are to develop pieces rapidly and harmoniously so that they are well coordinated, control the center, improve the safety of one's king, and create weaknesses on the opponent's side. In general, white can expect to obtain an advantage, and black is happy to obtain a position with equal chances. Players also try to get positions that fit their own style (e.g., strategic vs. tactical). Matters are obviously made more complex by the fact that both players try to frustrate each other's efforts. Importantly, opening knowledge can be seen as compiled search: the product of decades of research and practice by many individuals is compressed in a ready-to-use form that can be acquired fairly easily by players, who do not need to carry out these investigations again. Of course, we have here a similar process to the growth of scientific knowledge.

There is a substantial literature on chess, mostly on chess openings. One of the largest chess libraries in the world contains more than 50,000 books [27], so one can assume that many more have been written on this topic. Several encyclopedias of chess openings have been written, amongst which the work edited by Matanović and colleagues [21] has been the most influential. Considerable information about chess openings can also be obtained from chess databases [28] and chess playing computer programs [29]. In these media, chess experts recommend the best moves in a given position, mostly based on the outcome of previous games and the analysis of key positions in these games. This body of knowledge is called “chess theory,” although this is a misleading name. Unlike in science, “theory” in chess does not consist of a set of principles or laws that summarize and explain data, but is rather a catalogue of moves that have been played in competitive games, with an evaluation of each relevant branch of the game tree and sometimes a summary of the key strategic and tactical ideas. Chess theory also has a prescriptive value in that players tend to follow it as much as they can.

Departure from theoretical knowledge can happen for two main reasons, which cannot always be disentangled with certainty. First, and most commonly, in particular with weaker players, it can indicate lack of knowledge. A player plays a move that is so obviously inferior than the theoretical move(s) that it is not even mentioned by opening theory. Second, departure from theoretical knowledge can be deliberate and indicates that a player has come up with a new idea in a given position. Such a new move is referred to as a “theoretical novelty.” Novelties are an important weapon in a player's arsenal, as they bring the opponent into unknown territory. Novelties can be the product of home preparation, in particular with chess professionals and the recent availability of chess engines and computer databases. Novelties can also be the product of inspiration during a game. Note that the term “theoretical novelty” is neutral as to whether the new move is better than the previously known move(s) – establishing the validity of a new move can take several years of study by the chess community and hundreds of new games. The presence of novelties means that chess opening theory is constantly evolving: the evaluation of lines changes and lines that were considered important a few years ago are now falling into oblivion. Thus, knowledge of chess openings is relative, and refers to the knowledge of chess theory at the time a game was played. Chess opening theory in 2000 is not the same as chess opening theory in 1900, as more knowledge about the game has been acquired in between. This creates one complication: as chess theory is not static, part of the knowledge gets lost and rediscovering this knowledge would count as a “novelty”.

As a chess game consists of a sequence of moves, it is possible to determine both the total number of moves in a game (henceforth: *length*, counted in ply) and the number of theoretical moves played in a game (henceforth: *opening knowledge*, also counted in ply). (In chess as in several other board games, the term *move* is somewhat ambiguous and refers either to a pair of moves (one white and one black move in chess) or to a single piece movement. To avoid ambiguity when providing estimates of depth in chess, we will use the term *ply*, which refers to one white move *or* one black move.) The central methodological assumption of this paper is that the player who first departs from a theoretical opening line (*player of interest*, PI) knows the theory up to the point of rupture. For example, assume that in the position after 1.e4 e5 2.Nf3 Nc6, white plays 3.a4. Assuming that 3.a4 is a novelty, knowledge is 4 ply deep. Another possibility was to consider that the moves known by a player are the moves from the beginning of the game to the last theoretical move played by this player; this would decrease our estimates by one ply (i.e., in our example, 2. … Nc6 would not be considered as known by white). Note that the goal of the present paper is to estimate the amount of rote opening knowledge, rather than to evaluate the quality of the novelty and to assess problem-solving skills. Thus, there was no point in discarding part of the data (i.e., bad novelties).

In the following, we used a large database of games to infer the amount of knowledge of opening moves that players of different skill levels have. We first empirically show that there are important skill differences in the amount of opening knowledge that players have. Then, we assess whether color and relative skill played a role when departing from theory. Finally, combining these quantitative estimates with estimates of the number of master-level games that were computed by de Groot and Gobet [14], we speculate on the amount of opening knowledge that chess players of various skill levels have mastered.

## Materials and Methods

### Levels of expertise

To establish chess players' levels of expertise, we used the Elo rating [30]. The Elo rating is a normally distributed rating scale with a mean of 1500 and a standard deviation of 200 points. A player who is 200 points stronger than the opponent has a 75.8% chance of winning a game, and a 400 point difference translates into a 91.9% winning probability. Considering that experts are players with an Elo rating with 2000 points or higher [30], we assigned players to two levels of expertise (non-expert vs. experts) divided in four classes of 200 Elo points each. Two classes, *class B* (1600–1799) and *class A* (1800–1999), were subdivisions of the non-expert group, and two classes, *candidate masters* (2000–2199) and *masters* (2200–2399), were subdivisions of the expert group. The selected skill levels ensured that a sufficient number of games was used for each class and had the advantage that they occupied adjacent positions in the rating scale, which made comparisons easier.

### Selection and processing of games

The games used for the analysis were taken from Fritz 12 [29], a program commercialized by Chessbase, the world leader for chess software. Fritz 12 consists of a chess-specific interface and comprises both a suite of search engines and a database. It also provides a theoretical tree of openings, which reflects the up-to-date state of opening theory. The theoretical tree is a tree of all significant moves that were played or analyzed. A module makes it possible to compare the moves of a game to this tree. Fritz is commonly used by grandmasters, including world champions, for training purposes, and the theoretical tree plays an important role in this. Thus, we can be confident that the theoretical tree does indeed reflect current expert knowledge of openings.

The games contained in the database span all levels of expertise and cover more than 500 years of practice. There is no criterion preventing a game from entering the database. Admittedly, for a long time only experts' games were recorded in books. Yet, since the advent of internet and the possibility for tournament organizers to record all the games with easy-to-use software, currently most the competitive games are recorded, including a wide range of levels. To our knowledge, there is no bias in entering games in the database. In the current research, we favored recent games in order to have a sufficiently large number of non-experts games in the sample.

We selected all the games played in 2008. We then used a series of filters to ensure data quality. The first filter consisted in deleting the games for which no result was recorded. A second filter deleted games that lasted only one ply. Finally, with respect to expertise, a third filter selected the games wherein players' Elo ratings were between 1600 and 2399. The final sample consisted of 76,562 games. For each entry, we recorded the Elo rating of white and black, the length of the game (in ply), and the result of the game. For both white and black players, we used their Elo to determine the skill level.

### Evaluation of the amount of theoretical information

Applying Fritz 12's theoretical tree module in each game, we determined which of the two players (the PI) departed first from the theoretical prescription. To the six variables extracted directly from the game records (see above), we added five indicators about the PI by extracting the following information in each game: the Elo rating, the color (white vs. black, coded as +1 and −1, respectively), the skill level (Class B, Class A, Candidate masters, Masters) and the measure of opening knowledge (i.e., depth of the last theoretical ply played). We also extracted information about *relative skill* (weakest vs. best). Relative skill is a dichotomous variable resulting from the comparison of the Elo ratings of the two opponents. If the PI was the weaker (stronger) of the pair we assigned −1 (+1). The procedure was applied to the 76,562 games. By the end of the procedure, each record was thus made of 11 pieces of information (see Table 1).

**Table 1:** Composition of a record in the database. PI: Player of Interest (i.e., player first deviating from a theoretical opening).

Information about	Variable	Values/range
White player	Elo Rating	1600–2399
	Skill level	Class B, Class A, Candidate Masters, or Masters
Black player	Elo Rating	1600–2399
	Skill level	Class B, Class A, Candidate Masters, or Masters
PI	Elo rating	1600–2399
	Skill level	Class B, Class A, Candidate Masters, or Masters
	Color	White or Black
	Relative skill	Weakest (−1) or strongest player (+1) of the pair
	Opening knowledge	Depth of last theoretical move in the game (in ply)
Game duration	Length	Number of moves in the game (in ply)
Outcome	Result	Win for white (1), black (0) or draw (0.5)

## Results

Results are organized into three sections. The first section reports descriptive statistics. The main purpose of this section is to describe the main features of the games constituting the database. The second section examines the relationship between skill and sequences of theoretical moves. This section aims to establish the profile of each level of expertise. We first test the influence of potential confounding factors (color of play and relative skill) and then examine how sequence length varies as a function of the skill level of the PI. In the third section, we provide estimates of the amount of opening knowledge that players of different levels of skill hold. This section presents mathematical models based on the empirical data of the previous section. The ultimate objective is to move from empirical indicators of linear sequences to theoretical estimates of knowledge organized as a tree.

### Descriptive statistics

Descriptive statistics for the whole sample of 76,562 games are displayed in Table 2. A number of important results can be noted. First, the mean length of a chess game is *M* = 78.73 ply (*SE* = .12 ply), out of which 16.76 ply on average (*SE* = .02 ply) are theoretical (chess meaning). This implies that 21.29% of the moves played in a game by players in the 1600–2399 range are moves that belong to “chess theory.” As the average score indicates (*M* = .537), white gains the upper hand in a majority of games. Experts' intuition that white has a theoretical advantage is statistically supported, χ^2^(2, *N* = 76,562) = 1608.98, *p*<.01. This result could apparently be accounted for by the fact that the average Elo of white players is superior to that of black players. However, the small difference of 3.02 Elo between the groups, although statistically significant *t*(76,561) = 4.25, *p*<.01, is negligible. With such a difference, the probably of winning (or losing) for both players is the same as with a zero-difference, that is .05 (see the Table in section 2.1, p. 31, in Elo, 1978). Thus, this difference cannot account for the superiority of white. Nor is there any evidence that the fact that white players had a superior Elo rating reflect a selection bias in the database. With respect to skill level, the average Elo rating for both white and black is in the expert zone.

**Table 2:** Descriptive statistics for the whole sample. PI: Player of Interest (i.e., player first deviating from a theoretical opening).

Variable		Mean	SE
Elo rating	White	2112.81	.64
	Black	2109.79	.64
Knowledge		16.76	.02
Length		78.73	.12
Result		.537	.0015
PI Elo	Elo	2103.20	.65
PI color	White	49.33%	n/a
PI color	Black	50.66%	n/a
PI relative skill	Weakest	53.36%	n/a
	Best	46.64%	n/a

### Assessing opening knowledge

**Figure 1:**
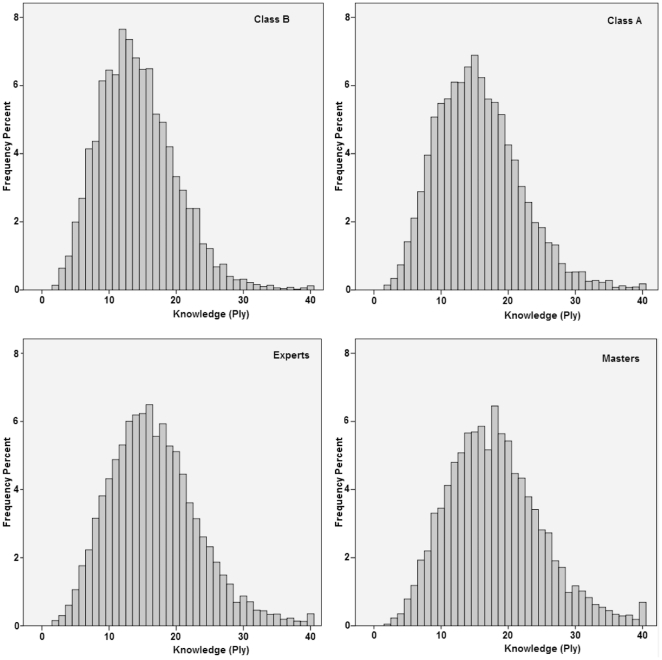
Frequency distribution of opening knowledge, for each skill level. With Fritz, all novelties at ply 40 and above (0.41% of the sample) are scored at ply 40 and are not shown in the histograms.

The distribution of the opening knowledge within each skill level is shown in Figure 1, and summary statistics are provided in Table 3. The charts in Figure 1 show that the peak of the distribution shifts towards the right as the level of expertise increases.

**Table 3:** Statistics describing the distribution of opening knowledge for each level of expertise.

Variable	Level of Expertise			
	Class B	Class A	Candidate Masters	Masters
N	5,019	15,737	29,881	25,925
Elo (SE)	1721.29 (.78)	1915.11 (.45)	2103.21 (33)	2291.31 (.35)
Mean knowledge (SE)	14.26 (.10)	15.58 (.11)	16.71 (.12)	18.01 (.12)
95% CI upper bound	14.10	15.48	16.63	17.93
95% CI lower bound	14.42	15.68	16.78	18.10
Median	14.00	15.00	16.00	18.00
Variance	32.68	38.76	43.30	48.34
SD	5.72	6.23	6.58	6.95
Skewness	0.68	0.64	0.59	0.55
Kurtosis	0.86	0.58	0.47	0.27

Before testing whether the amount of opening knowledge varies with the skill level of the PI, we checked for potential confounds. We first examined whether PI was playing white more often than black and whether she was the best or the weakest of the two opponents. Since weak players are expected to know less, we expected that the PI would be the weakest player. Strong players might occasionally come up with new moves early on, but they will also play novel moves after lengthy sequences of theoretical moves; thus, on average, they will show more theoretical knowledge. Since black is on the defensive and a mistake by black is more costly than a mistake by white, we also expected that the PI would play white. The color and relative skill frequencies for the PI are presented in Table 2.

Contrary to our expectations, black introduced the novelty more often χ^2^(1, *N* = 76,562) = 13.70, *p*<.01. Also, the departure from theory was introduced more often by the weakest player than by the best player χ^2^(1, *N* = 76562) = 345.89, *p*<.01. This result, which demonstrates that better players know more, is in full agreement with our expectations.

The amount of opening knowledge cannot surpass the total number of ply of a game, which implies that the amount of opening knowledge shown by a player is constrained by the length of the game. We tested whether opening knowledge and length are associated. Regressing length on opening knowledge yielded the following equation: *Length* = .331×*knowledge*+73.188 (Beta = .068), *F*(1, 76561) = 350.98, *p*<0.01, *MSE* = 1064.83. However, the amount of variance in length of the game accounted for by knowledge for is minimal (*r^2^*<.01).

An ANCOVA was carried out on opening knowledge with skill as the independent variable and length, color and relative skill as covariates. As predicted, the PI's skill level significantly affected opening knowledge, *F*(1, 76555) = 822.11, *p*<.01, *MSE* = 43.01, showing that opening knowledge varies as a function of skill. This is a crucial result showing that chess players accumulate static knowledge that guides them in the first phase of the game. The result that experts know more might seem trivial. Yet, beyond the fact that we offer the first quantification of opening sequence knowledge in chess, the result gains in importance when one considers that opening knowledge represents on average 21.29% of the length of a game. Table 3 shows the average number of ply known by each class of players. The fact that skill retains a significant effect when the variance of covariates [length, *F*(1,76555) = 290.56, *p*<.01, *MSE* = 43.01; color, *F*(1, 76555) = 18.71, *p*<.01, *MSE* = 43.01; and relative strength, *F*(1, 76555) = 358.50, *p*<.01, *MSE* = 43.01] is subtracted out illustrates the robustness of the main effect.

To further explore the relationship between opening knowledge and skill level, we carried out a post-hoc test. The data in Table 3 were entered in a regression with Mean Elo ratings as independent variable and opening knowledge (mean number of theoretical ply) as dependent variable. As expected, there is a strong relationship between skill and opening knowledge: Static Knowledge = (0.0065 * Elo)+3.0446, F(1, 2) = 2902.45, *p*<0.01, *MSE*<0.01. This equation accounts for 99% of the variance.

Finally, we note that the probability of playing a sequence of theoretical moves by each side randomly choosing a legal move or randomly choosing a master-game-like (mgl) move (see below) is negligible. Based on the estimates for legal moves (n = 32.3) and mgl moves (n = 1.76) given in [1], [14], the corresponding probabilities for Masters are (1/32.3)^18.01^ = 6.596×10^−28^, and (1/1.76)^18.01^ = 3.787×10^−5^, respectively.

### Estimating the amount of opening knowledge

This section will make use of the notion of *game tree* [31]. In graph theory, a game tree is a directed graph consisting of nodes (which denote the positions in the game) and edges or links (which denote the moves connecting two positions). In the case of chess, a constraint is that white and black play alternately. The *branching factor* is the number of edges going out of a node.

Using the average depth at which players deviate from known opening moves, we can estimate, for each skill level, the number of opening moves they know. For this, a few assumptions have to be made. We will assume uniform depth and constant branching factor. For the branching factor of the opponent, we will use two numbers. We first use the estimate proposed by de Groot, Gobet and Jongman [14], [32]. Based on a combination of empirical data and theoretical assumptions derived from information theory, these authors proposed that the branching factor is 2 (1 bit of information) from move 1 to move 20 (ply 40). However, it could be argued that this underestimates the real branching factor, as what can be considered as a master-likely move has changed with the availability of computers, which have shown that moves previously considered as non-playable actually are playable. To take this into consideration, we will consider another branching factor (n = 3).

In the following, we will make use of the notion of an *opening repertoire*. An opening repertoire is a set of openings that a player specializes in and normally plays. Typically, a player would prepare a repertoire for playing white and another for playing black. The amount of knowledge on chess openings is such that it is impossible to know many openings well, and, from a pragmatic point of view, it is preferable to play openings one has studied in detail. Chess players are advised to devote between 25 to 50% of their training time to develop and fine-tune their opening repertoire [33], [34].

### We consider two models

- **Model 1: One repertoire, player selects one move every time.** In this model, we assume that a player (P) has prepared one move to counter each of the opponent's (O) moves in the opening. We also assume a constant branching factor *n* for this opponent up to depth *d*, given by the data in Table 3. Thus, when playing white, P has selected one move, to which O has n alternatives. For each alternative, P has prepared one reply; for each of these replies, O has n possible alternatives. Thus, we have a tree where the branching factor is 1 at the odd levels and n at the even levels. For d = 1, the number of moves is 1. For d>1, the number of moves at level d is given by the exponential formula: n^d//2^, where//indicates division with rounding down. The total number of moves up to level d is given by 
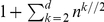
, where n is the branching factor. The same logic applies when P plays black, and the total number of nodes is 
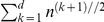
. The estimates are shown in Table 4, for n = 2 and n = 3. (Note that in this Table, the estimates of opening knowledge from Table 3 have been rounded.)

**Table 4:** Number of moves learned in an opening repertoire for white, assuming Model 1 and number opponent's moves (n) prepared being either two or three.

Skill level	Knowledge (in ply)	n	Number of moves learned		
			White	Black	Total
Masters	18	2	1,533	2,044	3,577
Candidate masters	17	2	1,021	1,532	2,553
Class A	16	2	765	1,532	2,297
Class B	14	2	381	508	889
Masters	18	3	39,364	59,046	98,410
Candidate masters	17	3	19,681	39,363	59,044
Class A	16	3	13,120	19,680	32,800
Class B	14	3	4,372	6,558	10,930

How large is a number like 98,410? A recent study of chess players' autobiographic memory [35] sheds light on this question. The study showed that two strong masters (2550 and 2500 Elo, respectively) were able to recognize positions as belonging or not to their own games almost perfectly (97.2% and 89.1%, respectively), suggesting that they had nearly fully memorized the moves of these games. At the time of the experiment, Chessbase had 403 games and 423 games for these two players, respectively. Assuming an average length of 40 moves (80 ply) and that all sequences are different, and ignoring games that were not in the database (probably at least as many games as those available), this would suggest that they had memorized at least 31,337 ply (403×0.972×80) and 30,151 ply (423×0.891×80), respectively. (This is a slight overestimate, since some of the positions early in the game (e.g., after 1.e4) recur several times.) Thus, 98,410 is about the same as three times the number of ply these players had memorized in their own games according to our estimations, which seems plausible.

- **Model 2: Two repertoires, one own alternative.** Systematically playing the same move in the same position, as is the case with P so far, has the obvious disadvantage that play is predictable. Opponents can take advantage of this by preparing new moves, which will force P out of his comfort zone. One way of countering this approach is, from the point of view of P, to play different openings. Thus, P will still prepare one move per reply, but will use two repertoires. For example, one repertoire could lead to more tactical positions, and one repertoire could lead to more strategic positions. With this approach, the number of moves learned is simply the double of the numbers of the relevant column of Table 4.

Of course, using two repertoires with a single alternative still leaves P open to O's coming up with novelties. Ideally, one would like to prepare several moves in each position. However, the number of moves to learn rapidly becomes unwieldy given the exponential nature of the chess game tree. With balanced trees (same number of alternative for white and black), the number of moves at level d is given by the exponential formula: n^d^, and the total number of moves up to level d is given by 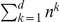, where n is the branching factor. With n = 2, where this formula simplifies to: n^(d+1)^ – 2, the number of moves is already unrealistic. Using the depths provided in Table 3, a class A player would have to learn 78,474 moves for a repertoire for black and white, and a Master would have to learn 1,055,865 moves.

We can consider our estimates based on model 1 with a branching factor of 2 as a lower bound, and those based on a full balanced tree (n^d^) with a branching factor of 2 as an upper bound. We will focus our discussion on intermediates estimates, those provided by model 1 with a branching factor of 3.

## Discussion

In this article, we were interested in comparing the amount of opening knowledge acquired by players of four skill levels: two levels below the expert cut-off and two levels above it. A large number of games were analyzed with a chess program able to pinpoint, for each game, the point where players departed from the theoretical prescription. We used these results to estimate the amount of opening knowledge learned by players.

While it was expected that opening knowledge would increase with players' skill level, the main contribution of this paper is to have provided quantitative estimates of this increase. We have shown that strong chess players have a deep knowledge of the first phase of the game. This result suggests that chess players rely on previous knowledge for many moves and postpone the start of real thinking until their opening knowledge is exhausted. Only then must they come up with original answers to their opponent's moves. Since players devote a considerable part of their training to learn predetermined sequences of moves in the openings, and given the number of hours they devote to chess, some of the positions occurring in openings will have been seen many times. Hence, there will have been many opportunities to create chunks [17] and templates [16] for them. Templates would encode key positions in a given opening, especially those where the opponent has several choices, thus adding to the variability necessary for creating slots. They would provide information about possible moves and maneuvers, and thus help organize the information about theoretical moves. Importantly, templates are at the intersection between monochrestic and polychrestic knowledge. Since they are flexible, templates might be used to organize moves around strategic or tactical themes facilitating the long-term storage of new moves. This hypothesis reinforces the idea that templates are at the core of chess knowledge.

It is interesting to compare our estimates with that provided by Charness [20] (1,200 opening sequences up to ply 20, i.e., 24,000 moves), which is about one fourth of our intermediate estimate. Note that Charness focused on main lines, but, at professional level, a player must know a large number of secondary lines as well. Note also that, with the advent of computer programs playing chess at a high level and databases with substantial information about openings, the pressure is much higher to memorize the detail of openings than when Charness wrote his chapter – players who do not do it put themselves at a disadvantage compared to opponents that do.

Monochrestic knowledge and polychrestic knowledge differ in one important way. Monochrestic knowledge is linked to the time course of moves and its application is contingent to the exact opening position. By contrast, polychrestic knowledge can be used with several opening systems as it refers to patterns of pieces constrained spatially but not temporally. Regardless of the history of the game, a spatial pattern can be recognized. These two types of knowledge also share an important similarity: considerable amounts of them are needed for reaching master level. Putting together the estimated amounts of opening knowledge (∼100,000 moves) and chunk knowledge (∼300,000 chunks [24]) provides theoretical reasons why several years of hard work are a necessary condition for becoming a master [17], [36].

On average, opening knowledge increased by about 1.25 ply from one skill level to the next (see Table 3). As we delve more into the game, the tree of possibilities expands exponentially. Thus, a constant increase in mastery of opening moves, as measured by the difference in number of ply, requires increasingly more knowledge. For example, using model 2, moving from class A to candidate master required learning 26,000 new moves, while moving from candidate master to master required learning 39,000 new moves. This is a powerful illustration that the acquisition of expertise follows diminishing returns and increase in performance follows a power law [37]. This also means that small differences in learning rate, perhaps due to genetic differences [38], will have large consequences, given the number of chunks and moves that have to be acquired. That there exist considerable individual differences in the time necessary to become a master has been shown in a study about the practice patterns of Argentinean chess players, in which some individuals needed 8 times longer than others [36].

Our study has several limitations. First, the central assumption is that the departure from theoretical prescription marks the exact limit of players' knowledge. But it is possible that some players played theoretical moves without knowing it, just by applying general heuristics. However, as suggested by the estimates provided earlier about the likelihood of finding a theoretical sequence by chance (e.g., 3.787×10^−5^ by sampling from master-game like moves), we do not expect this effect to be large, even if we cannot rule it out completely. Regularly finding theoretical moves without prior knowledge would imply that players play near perfectly in complicated situations, but we know that players of similar skill levels commit multiple errors during a game [39].

Second, several assumptions had to be made in order to provide quantitative estimates of the amount of opening knowledge. These include the assumption of uniform depth and of a constant branching factor. However, players almost certainly study openings at different depths; for example, they would particularly study tactical lines at great depth, sometimes until the endgame. In addition, the branching factor tends to be high in the first opening moves and low after – often only 1 move. It is also likely that there are individual differences in the way players study openings. We take the view that, at a first approximation, the opposite effects of these assumptions tend to cancel each other.

Third, the method with which Fritz builds its database of openings and evaluates the novelty of moves includes idiosyncratic decisions (e.g., limit to 40 ply). In addition, variations in the cut-off date for a game inclusion as well as omission of some games in the opening database mean that, if anything, our analysis underestimates the amount of opening knowledge. While these limits are real, it should be pointed out that chess is one of the few domains of expertise that allow quantitative estimates of knowledge to be made. Consider, for example, how difficult it would be to quantify the knowledge acquired by a philosopher or musician.

Further research might test the generality of our conclusions, for example by analyzing opening knowledge in other games, such as Go and draughts. Another possibility for validating our results is to examine the actual opening repertoire of selected players. In the past, repertoires were written down in notebooks or files, and they are now typically stored in computer databases; there is thus objective evidence that could be used to test our estimates. Finally, our estimates assumed that all skill levels had prepared the same number of moves against the opponent's moves. In other words, the branching factor was constant across skill levels. A possibility is that, as skill increases, differences emerge in both depth and breadth. Thus, further research might investigate models where both dimensions vary.

In general, our results add further support to Holding's [2] view emphasizing the role of declarative knowledge in high levels of expertise; indeed, they support the importance of monochrestic and rote knowledge – undoubtedly with understanding. In this respect, they provide an important qualification on recent claims that expertise can be explained mostly by unconscious and intuitive processes [40]–[43]. A similar role for rote and declarative knowledge is present in many domains of expertise, such as science and law, and it is important for further research to understand how this knowledge is acquired. This being said, chess and other domains of expertise also require the acquisition of perceptual, intuitive and procedural knowledge, as has been amply documented in the literature [44], [45]. Becoming an expert is a complex process, and thus it is not surprising that it requires acquiring multifarious types of knowledge.
